# A bi-institutional multi-disciplinary failure mode and effects analysis (FMEA) for a Co-60 based total body irradiation technique

**DOI:** 10.1186/s13014-021-01894-3

**Published:** 2021-11-19

**Authors:** Shahbaz Ahmed, Todd Bossenberger, Adrian Nalichowski, Jeremy S. Bredfeldt, Sarah Bartlett, Kristen Bertone, Michael Dominello, Mark Dziemianowicz, Melanie Komajda, G. Mike Makrigiorgos, Karen J. Marcus, Andrea Ng, Marvin Thomas, Jay Burmeister

**Affiliations:** 1 Department of Oncology, Wayne State University School of Medicine, Detroit, MI USA; 2 Gershenson Radiation Oncology Center, Karmanos Cancer Center, Detroit, MI USA; 3 Dana Farber/Brigham and Women’s Cancer Center, Harvard Medical School, Boston, MA USA

**Keywords:** FMEA, TBI, Bi-institutional, Multidisciplinary, Risk assessment, Risk estimation, TG-100, Quality management

## Abstract

**Background:**

We aim to assess the risks associated with total body irradiation (TBI) delivered using a commercial dedicated Co-60 irradiator, and to evaluate inter-institutional and inter-professional variations in the estimation of these risks.

**Methods:**

A failure mode and effects analysis (FMEA) was generated using guidance from the AAPM TG-100 report for quantitative estimation of prospective risk metrics. Thirteen radiation oncology professionals from two institutions rated possible failure modes (FMs) for occurrence (O), severity (S), and detectability (D) indices to generate a risk priority number (RPN). The FMs were ranked by descending RPN value. Absolute gross differences (AGD) in resulting RPN values and Jaccard Index (JI; for the top 20 FMs) were calculated. The results were compared between professions and institutions.

**Results:**

A total of 87 potential FMs (57, 15, 10, 3, and 2 for treatment, quality assurance, planning, simulation, and logistics respectively) were identified and ranked, with individual RPN ranging between 1–420 and mean RPN values ranging between 6 and 74. The two institutions shared 6 of their respective top 20 FMs. For various institutional and professional comparison pairs, the number of common FMs in the top 20 FMs ranged from 6 to 13, with JI values of 18–48%. For the top 20 FMs, the trend in inter-professional variability was institution-specific. The mean AGD values ranged between 12.5 and 74.5 for various comparison pairs. AGD values differed the most for medical physicists (MPs) in comparison to other specialties i.e. radiation oncologists (ROs) and radiation therapists (RTs) [MPs-vs-ROs: 36.3 (standard deviation SD = 34.1); MPs-vs-RTs: 41.2 (SD = 37.9); ROs-vs-RTs: 12.5 (SD = 10.8)]. Trends in inter-professional AGD values were similar for both institutions.

**Conclusion:**

This inter-institutional comparison provides prospective risk analysis for a new treatment delivery unit and illustrates the institution-specific nature of FM prioritization, primarily due to operational differences. Despite being subjective in nature, the FMEA is a valuable tool to ensure the identification of the most significant risks, particularly when implementing a novel treatment modality. The creation of a bi-institutional, multidisciplinary FMEA for this unique TBI technique has not only helped identify potential risks but also served as an opportunity to evaluate clinical and safety practices from the perspective of both multiple professional roles and different institutions.

**Supplementary Information:**

The online version contains supplementary material available at 10.1186/s13014-021-01894-3.

## Background

Total body irradiation (TBI) is a specialized radiotherapy technique for cancers involving the entire body, i.e., leukemias, lymphomas, myeloma, or other hematological malignancies [1]. The treatment is typically delivered either with a dedicated facility or as a modified application of radiotherapy equipment used for routine treatments [1, 2]. The uniqueness of TBI dose delivery demands a specialized quality management (QM) program.

Until recently, radiotherapy QM programs have focused primarily on assessing the functional performance of equipment. As suggested by guidelines from various organizations (i.e., AAPM, ACR, ACMP, IAEA, ESTRO, IEC, and ISO), it is always desirable to check and document every measurable parameter. However, given the extent and variety of radiotherapy techniques in clinical practice, this approach is not practical in terms of effective resource utilization. Therefore, the guidelines of the AAPM TG-100 report recommend a new framework for the design of the QM program [3].

This framework is mainly based on introducing prospective QM techniques. The Failure Mode and Effects Analysis (FMEA) is one such technique recommended by TG-100 [3]. Current literature exists on the application of the FMEA for intensity-modulated radiotherapy (IMRT) as well as various special procedures [4–14]. The FMEA technique has also been applied to acceptance and commissioning processes, clinical reference dosimetry, and radiobiological research with small animal irradiators [15–19]. Such an analysis is primarily helpful in the QM of novel processes and treatment techniques as well as for specialized radiotherapy procedures, such as TBI, which are already being performed.

Two investigators have recently published quality improvement analyses specific to TBI. Kim et al. presented a retrospective analysis of TBI treatments by analyzing the data from an incident learning system [20]. Shen et al. recently shared their experience using FMEA for total marrow irradiation (TMI), a more narrowly-targeted modification of TBI [21]. The authors of this study reported that a second FMEA analysis performed for high-risk failure modes (FMs) 1 year following the initial FMEA improved their QM program. While these two analyses have provided insight into FMs in TBI, there is a paucity of data examining whether the identified FMs may be generalizable between institutions. Furthermore, FMs specific to dedicated TBI equipment have not been reported yet.

This study describes the development and evaluation of an FMEA for a novel dedicated Co-60 based TBI delivery unit. It represents the first bi-institutional, multi-disciplinary FMEA for the TBI technique and should also be applicable to other forms of TBI/TMI/TMLI [22]. We will discuss the advantages and difficulties involved in an inter-institutional, inter-professional FMEA.

## Materials and methods

Karmanos Cancer Institute (KCI) and Dana Farber/Brigham and Women’s Cancer Center (DFBW) have implemented a commercial dedicated Co-60 based TBI irradiator (GammaBeam 500 by Best Theratronics, Inc., Kanata, ON, Canada). The clinical commissioning of this unit has been previously described [23]. Two multi-disciplinary teams were formed for the FMEA, one at each institution (KCI and DFBW). The KCI team included three medical physicists (MPs), two radiation oncologists (ROs), and two radiation therapists (RTs) whereas the DFBW team included two participants for each of these disciplines. The specific individuals most heavily involved with the respective TBI programs were chosen to participate and all participants were familiar with the TG-100 methodology for FMEA [3].

Some additional information is provided here regarding the processes involved in treating patients with TBI on this unit, both to help the reader better understand the equipment and techniques and to allow a comparison to other treatments and modalities. The two institutions have similar treatment programs with only relatively minor operational differences. The major differences between these programs are in the in-vivo dosimetry and dose calculation procedures. While DFBW performed in-vivo dosimetry during the initiation of the treatment program, these have been discontinued. KCI performs in-vivo dosimetry for all ports for all patients. At both facilities, in-vivo dosimetry is/was performed only for part of the treatment delivery time. This time is manually subtracted from the prescribed treatment time for delivery of the remainder of the treatment. Since commercial treatment planning systems are not designed to handle such large SSDs, dose calculations are performed using in-house developed techniques. KCI uses a Monte Carlo based system to calculate the relative dose distribution [23] and both a spreadsheet system and redundant manual calculations for the absolute dose. DFBW uses a correction based manual calculation and then a different correction-based spreadsheet calculation as a secondary check. The spreadsheet formulas at both institutions are locked and password protected. There are two flattening filters (thick and thin) which are modeled in the MC dose calculation system at KCI. Compensators are created manually from regular geometric shapes of leaded polyethylene. At KCI, in-vivo dosimetry is performed at 12 points (head, suprasternal notch, umbilicus, knees, ankles, and under lung blocks for both AP and PA fields) for multifraction treatments and at the umbilicus for AP and PA fields for single 2 Gy fraction treatments. Doses are compared to both manual and MC calculations at KCI and to both correction-based calculations at DFBW. Image data is managed within a commercial imaging software system provided by Best Theratronics, Inc. Both facilities use the Aria electronic medical record system but not in a mode which interfaces with the treatment delivery system of the GammaBeam 500 system. As such, all data are manually entered into the GammaBeam 500 console and subsequently into Aria.

**Fig. 1:**
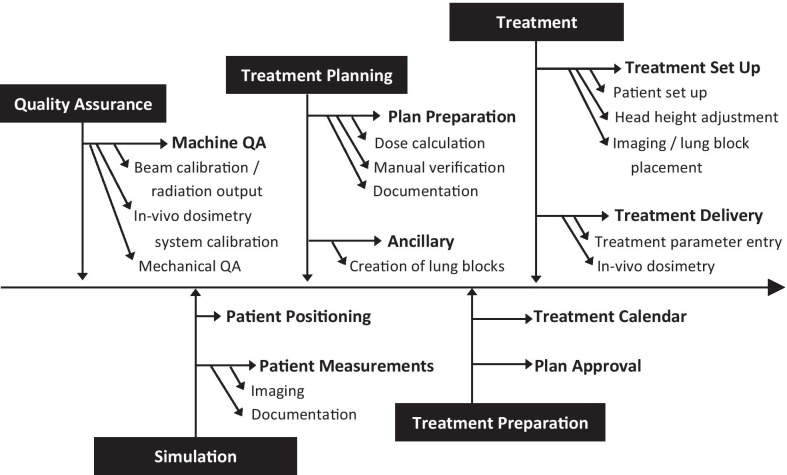
Process map for the Co-60 based TBI technique

The process mapping and identification of potential FMs were performed jointly. Participants from all disciplines and both institutions were involved to identify the maximum range of possible FMs, as recommended by the TG-100 guidelines [3]. The list of FMs was then sent to the manufacturer of the TBI unit in an attempt to identify any additional potential FMs not already identified by the staff at the two treatment facilities. The entire treatment procedure was divided into five processes, namely; logistics, simulation, planning, delivery, and quality assurance (QA) with a total of nine sub-processes as depicted by the process map shown in Fig. 1.

The indices of occurrence (O), severity (S), and detectability (D) were rated separately by the participants in each team on a 1–10 scale as described in Table 2 of the TG-100 report [3]. As recommended, the FMEA was performed with the assumption that there were no specific QA/QC (quality control) measures in place and that O and D should be based entirely on checks that are inherent in routine clinical processes downstream [3]. A risk priority number (RPN) was then obtained for each FM as the product of these three indices and the FMs were ranked with respect to both RPN value and severity. Averaged FMEA index data were calculated, resulting in the following twelve cohorts:

- One (1) FMEA by all participants from both institutions (named as Aggregate-FMEA)
- Two (2) FMEA by all participants from one institution (named as KCI-FMEA and DFBW-FMEA respectively)
- Three (3) FMEA, each with ratings from participants with the same specialty at both institutions (named as MP-FMEA, RO-FMEA, and RT-FMEA).
- Six (6) FMEA, each with ratings from participants with the same specialty and belong to the same institution (named as KCI-MP-FMEA, KCI-RO-FMEA, KCI-RT-FMEA, DFBW-MP-FMEA, DFBW-RO-FMEA, DFBW-RT-FMEA)

The RPN values for averaged FMEAs were calculated by multiplying the mean O, S, and D values. The standard deviations (SDs) in individual indices are summed in quadrature for getting the SD in RPN.

To provide an overall comparison for the various FMEAs listed above, a single matrix is required that could represent ratings for all the FMs. We chose to report minimum, maximum, median, mean of means (MoM), 1st and 3rd quartiles for O, S, D, and RPN for all the FMs in each of the 12 FMEAs.

A pair-wise comparison was performed for similarities and differences between institutions and specialties as per following comparison pairs (CPs);

- Inter-institutional (i.e. KCI vs DFBW)—1 CP
- Inter-professional (i.e. MP vs RO, RO vs RT, RT vs MP)—3 CPs
- Intra-institutional inter-professional (i.e. KCI-RO vs KCI-RT and so on)—6 CPs with 3 for each institution
- Inter-institutional intra-professional (i.e. KCI-vs DFBW-MP and so on)—3 CPs, 1 for each specialty

Top 20 ranked FMs (ranked by RPN) were compared using the Jaccard index (JI) defined as below;

$$JI\left( {A,B} \right) = \frac{{\left| {A \cap B} \right|}}{{\left| {A \cup B} \right|}} = \frac{{\left| {A \cap B} \right|}}{{\left| A \right| + \left| B \right| - \left| {A \cap B} \right|}}$$

where A and B are the sets of top 20 FMs in A-FMEA and B-FMEA respectively. $A \cap B$ denotes the number of common FMs in sets A and B. The higher the number of common FMs in two cohorts, the higher the JI value will be.

Similarly, the 13 CPs were analyzed for the number of FMs with complete data (n). Absolute gross differences (AGD) in RPN values were calculated and evaluated for comparison and contrast. The absolute difference measured between the RPN values (not from the differences in the individual indices) is termed the AGD. The range, mean, SD, and median of AGD in each CP was calculated.

A prioritization value for RPN as well as O, S, and D can be chosen for identifying high-priority FMs for evaluation at the first stage. In subsequent stages, the remaining set of FMs undergoes the same process until all FMs are evaluated for necessary actions. This stage-by-stage appraisal of FMEA based on such a prioritization helps in a smooth translation of changes into the QM program [3]. Here, we introduce an efficient way of prioritizing the FMs based on a plot of the cumulative number of FMs versus mean ratings. As shown in Fig. 4, for a desired number of FMs (on the x-axis) to be prioritized, a prioritization value can be determined on the y-axis.

The most frequently occurring (high O-rating), severe (high S-rating), the most difficult-to-detect (high D-rating), and high RPN FMs in all twelve FMEA spreadsheets are also discussed for inter-institutional and inter-professional comparison. FMEA reproducibility has previously been evaluated in the literature [21, 24]. To provide a sample set of data to evaluate the reproducibility of the risk assessment presented here, the FMEA was repeated by the KCI physicists approximately 2 years after the initial FMEA. These time points represent approximately 2 and 4 years following clinical implementation of the equipment and processes evaluated.

## Results

A total of 87 possible FMs were identified for the entire process. The treatment process had the maximum number of identified FMs i.e. 57 (setup: 31, delivery: 26), followed by QA: 15, planning: 10 (preparation of initial plan: 6, ancillary: 4), simulation: 3, and logistics: 2 FMs. A total of 66 FMs were completely evaluated by all participants from DFBW whereas only 51 were completely evaluated by KCI participants. Combined, 46 FMs had complete evaluation data from all participants at both institutions. However, as we note from Table 3, at the minimum, 71 FMs were rated by at least one individual for any CP. MPs rated the maximum FMs (86), whereas some ROs and RTs did not rank some or all indices for a few FMs due to their inability to accurately assess these indices. All data on individual and averaged FMEAs are given in “Supplementary Appendix 1” for reference.

Individual O, S, and D ratings for most participants spanned the full scale (1–10). However, ranges as low as 1–4 are also observed for some participants. Maximum RPN values ranged from 90 to 420 for individual raters.

**Fig. 2:**
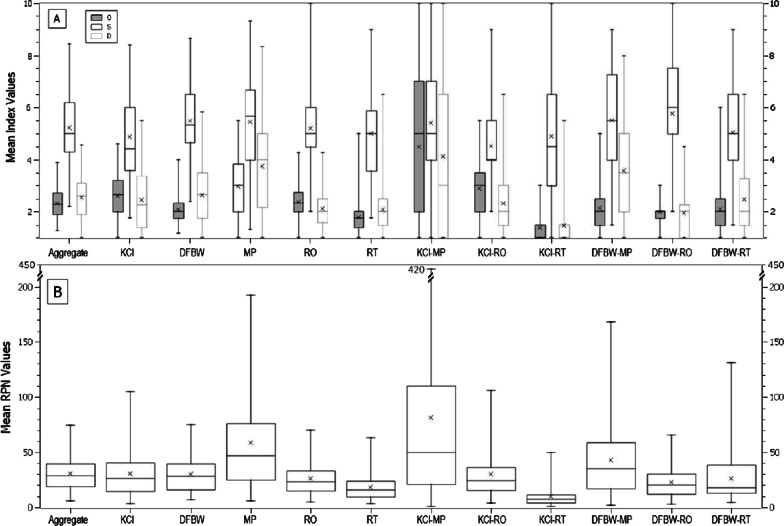
Range and extent of the data in a box and whisker plot for various FMEAs

Figure 2 represents the range and extent of the data obtained for the 12 averaged FMEAs on a box and whisker plot (whiskers show minimum and maximum, whereas the box show 1st, and 3rd quartile along with median, and the crossed datapoint shows the MoM for mean O, S, D, and RPN). We observe that the KCI-MPs generated the highest MoM (i.e. 81.6) and maximum (i.e. 420) for RPN scores compared to the rest of the participants. KCI-RTs generated the lowest MoM (i.e. 9.6) and maximum (i.e. 50) for RPN values, reflecting the inter-professional differences within an institution.

**Fig. 3:**
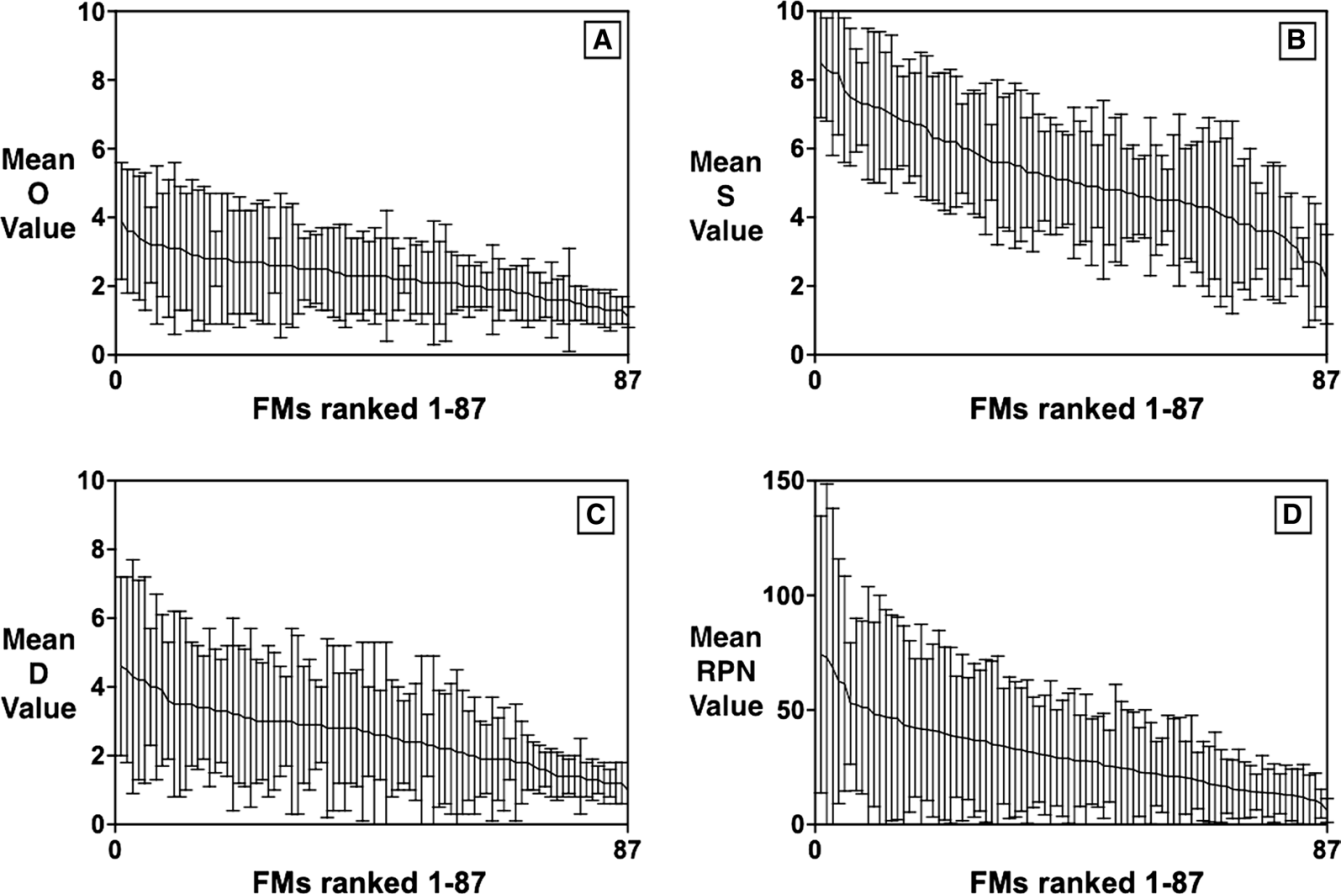
Statistical representation of mean O, S, D, and RPN data (in A, B, C, and D, respectively) for 87 FMs in descending order for Aggregate-FMEA [vertical bars show SD]

The extent of the aggregate-FMEA data is also illustrated in Fig. 3 which plots mean and SD for O, S, D, and RPN metrics in descending order. In general, participants tended to rate S values highest (8.5, SD:1.6) compared to D (4.6, SD:2.6) and O (3.9, SD:1.7) values. A similar trend exists for the range of mean index ratings in averaged FMEAs, with S having the maximum range (2.2–8.5), followed by D (1.0–4.6 [low ‘detectability’ values show that the FMs were more easily detectable]) and O (1.3–3.9), respectively. It is, however, to be noted that the SD is higher for RPN as compared to the individual index ratings.

**Fig. 4:**
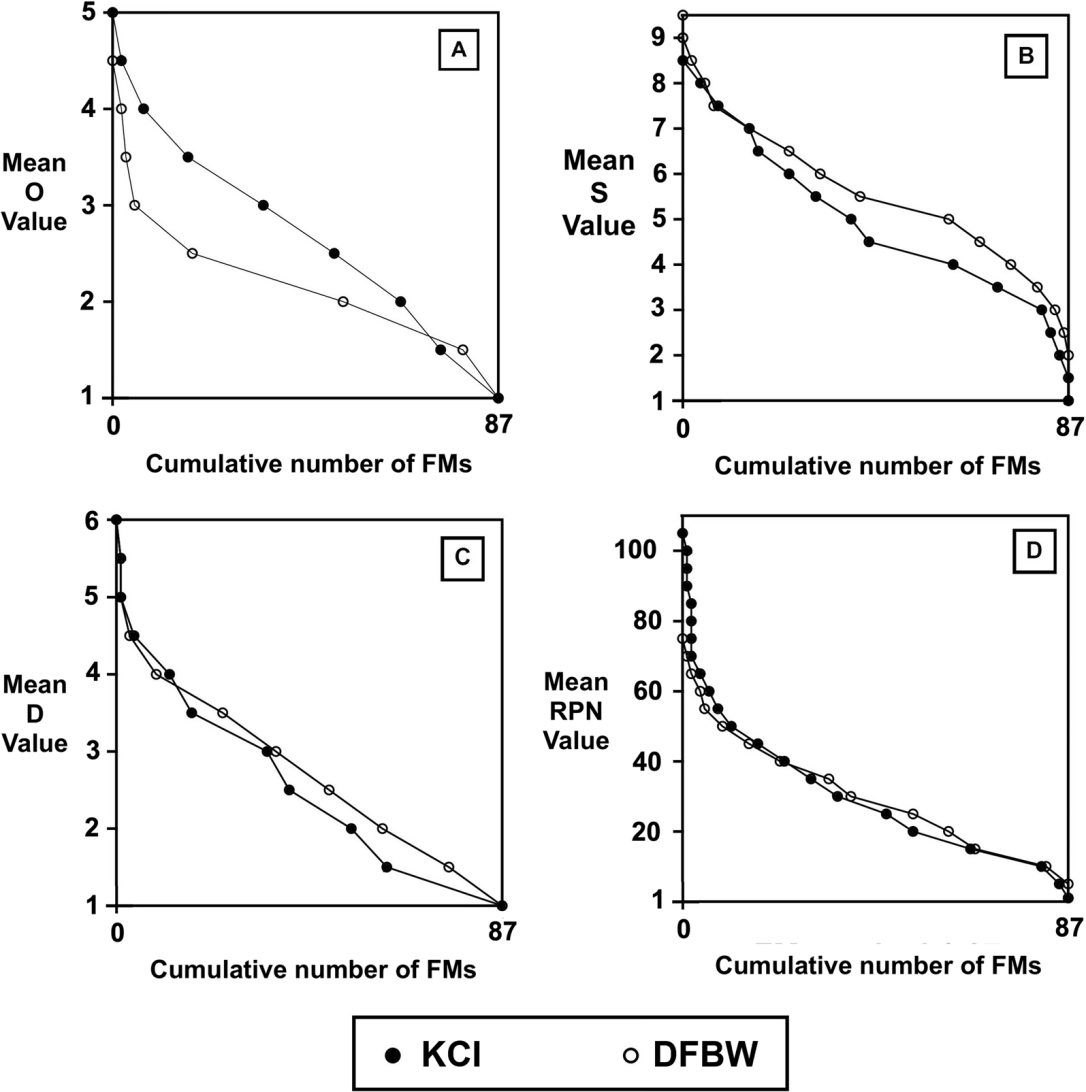
Cumulative number of FMs versus the overall mean value of O, S, D, and RPN (in A, B, C, and D, respectively)

Figure 4 shows the cumulative number of FMs corresponding to a particular rating or RPN value and allows visualization of differences between the two facilities. The top 20 FMs (ranked by RPN) were chosen for the first stage of implementation of our FMEA findings. This corresponds to an RPN prioritization value of 41 and 43 for DFBW and KCI, respectively. This prioritization value was arbitrarily chosen but could be chosen based on a particular RPN cutoff value or potentially by the slope of this curve. This prioritization roughly corresponds to 1/4 of the total number of FMs (i.e. 20 out of total 87 FMs). Table 1 presents the 20 highest ranking FMs for each institution for all groups ranked by total RPN and by Severity index. As observed in Table 1 and Fig. 4, the most pronounced difference between the mean RPN rankings for the two institutions is at the upper end of the RPN range.

**Table 1:** The top 20 FMs ranked by mean RPN and ‘S’ scores for each institution

Rank	FM#	Process description	Step description	Failure mode	Effects	O	S	D	RPN
**A: K C I – F M E A (7 scorers: 3 MPs, 2 ROs, 2 RTs) ranked wrt mean RPN scores**									
1	54	Treatment	Delivery	Patient moves after setup (with lung blocks)	Incorrect dose delivered	4.6 (2.1)	6.0 (0.6)	3.8 (1.9)	104.9 (72)
2	16	Treatment	Setup	Setup error (significant wrt tx field)	Incorrect dose delivered	3.3 (2.3)	5.8 (1.8)	4.8 (3.3)	88.8 (92.4)
3	8	Planning	Preparation of initial plan	Calculation error	Incorrect dose calculation	4.4 (3.1)	7.6 (2.0)	2.0 (0.6)	66.9 (54.9)
4	80	QA	Machine QA	Couch is accidentally shifted after morning QA (shift insignificant wrt tx field)	Incorrect dose delivered	4.3 (2.6)	3.3 (0.8)	4.8 (1.5)	65.6 (47.9)
5	49	Treatment	Delivery	Program wrong tx time	Incorrect dose delivered	3.0 (2.1)	7.0 (2.7)	3.0 (1.9)	63.0 (64.1)
6	53	Treatment	Delivery	Patient moves after setup (significant move wrt tx field) (no lung blocks)	Incorrect dose delivered	3.6 (1.9)	4.2 (0.4)	4.0 (1.8)	60.5 (41.7)
7	64	Treatment	Delivery	Used prone time for supine position or vice versa	Incorrect dose delivered	3.4 (2.1)	4.4 (0.5)	4.0 (3.3)	59.8 (61.4)
8	52	Treatment	Delivery	Timer malfunction	Incorrect dose delivered	2.2 (1.2)	7.4 (2.3)	3.4 (1.4)	55.4 (40.7)
9	18	Treatment	Setup	Missing lung blocks (one fraction)	Incorrect dose delivered	3.0 (1.7)	4.4 (0.8)	4.0 (3.2)	52.8 (52.0)
10	5	Sim	Pt positioning	Incorrect documentation	Incorrect patient setup	3.6 (1.6)	3.0 (1.1)	4.8 (3.2)	51.8 (46.2)
11	12	Planning	Ancillary	Incorrect lung block design/size	Lung overdose / target underdose	4.0 (3.0)	5.8 (2.8)	2.2 (1.0)	51.0 (50.5)
12	63	Treatment	Delivery	Forget to flip to supine after prone field	Incorrect dose delivered	2.0 (1.1)	6.8 (1.7)	3.6 (3.4)	49.0 (54.7)
13	3	Sim	Pt measurements	Incorrect mx	Incorrect dose calculation	3.8 (2.4)	4.0 (0.6)	3.2 (1.6)	48.6 (39.9)
14	11	Planning	Preparation of initial plan	Corrupt spreadsheet	Incorrect dose calculation	3.0 (1.6)	8.0 (0.8)	2.0 (0.8)	48.0 (33.0)
15	69	Treatment	Delivery	Image from wrong date is used for alignment verification	Lung blocks positioned incorrectly	2.8 (1.0)	4.2 (1.0)	4.0 (3.0)	47.0 (40.8)
16	65	Treatment	Delivery	Patient has too many blankets covering them	Change in dose distribution	3.8 (2.7)	3.6 (1.0)	3.4 (2.2)	46.5 (47.1)
17	4	Sim	Pt measurements	Incorrect documentation	Incorrect dose calculation	3.8 (2.7)	3.8 (1.5)	3.2 (1.7)	46.2 (45.0)
18	81	QA	Machine QA	Couch is accidentally shifted after morning QA (shift significant wrt tx field)	Incorrect dose delivered	3.3 (1.5)	5.0 (1.6)	2.8 (1.5)	44.7 (34.5)
19	68	Treatment	Delivery	Images saved under wrong patient	Inability to localize lung blocks, potentially incorrect dose delivered	3.0 (1.3)	4.2 (1.7)	3.4 (3.3)	42.8 (48.9)
20	6	Planning	Preparation of initial plan	Miscommunication of Rx	Incorrect dose or fractionation	1.6 (0.5)	7.0 (2.3)	3.6 (3.4)	40.3 (42.0)

Rank	FM#	Process description	Step Description	Failure mode	Effects	O	S	D	RPN
**B: K C I—F M E A (7 scorers: 3 MPs, 2 ROs, 2 RTs) ranked wrt mean ‘S’ scores**									
1	42	Treatment	Setup	Flattening filter falls on patient	Patient injury	1.8 (1.6)	8.4 (2)	1.2 (0.4)	18.1 (17.7)
2	47	Treatment	Delivery	Wrong patient	Incorrect dose delivered	1.4 (0.5)	8.2 (2.1)	1.2 (0.4)	13.8 (7.6)
3	11	Planning	Preparation of initial plan	Corrupt spreadsheet	Incorrect dose calculation	3.0 (1.6)	8.0 (0.8)	2.0 (0.8)	48.0 (33.0)
4	79	QA	Machine QA	Source stuck and emergency shutter not operational	Patient overdose	1.0 (0.0)	8.0 (2.8)	3.7 (3.8)	29.3 (31.9)
5	8	Planning	Preparation of initial plan	Calculation error	Incorrect dose calculation	4.4 (3.1)	7.6 (2.0)	2.0 (0.6)	66.9 (54.9)
6	19	Treatment	Setup	Missing lung blocks (all fractions)	Incorrect dose delivered	1.4 (0.8)	7.6 (2.6)	3.2 (3.5)	34.0 (43.5)
7	39	Treatment	Setup	Flattening filter not inserted	Incorrect dose delivered	1.4 (0.5)	7.6 (2.2)	1.6 (0.8)	17.0 (11.5)
8	56	Treatment	Delivery	Source stuck	Incorrect dose delivered/staff exposure	1.6 (0.5)	7.6 (2.3)	1.2 (0.4)	14.6 (8.0)
9	52	Treatment	Delivery	Timer malfunction	Incorrect dose delivered	2.2 (1.2)	7.4 (2.3)	3.4 (1.4)	55.4 (40.7)
10	45	Treatment	Setup	Motor run away causing head to contact patient	Patient injury	1.4 (0.5)	7.4 (2.2)	1.2 (0.4)	12.4 (7.0)
11	28	Treatment	Setup	Couch not at treatment height for multiple fx (large change > 5 cm)	Incorrect dose delivered	2.0 (1.5)	7.2 (2.3)	1.4 (0.5)	20.2 (18.3)
12	49	Treatment	Delivery	Program wrong tx time	Incorrect dose delivered	3.0 (2.1)	7.0 (2.7)	3 (1.9)	63.0 (64.1)
13	6	Planning	Preparation of initial plan	Miscommunication of Rx	Incorrect dose or fractionation	1.6 (0.5)	7.0 (2.3)	3.6 (3.4)	40.3 (42.0)
14	13	Planning	Ancillary	Incorrect compensator	Incorrect dose delivered	2 (1)	7.0 (2.0)	2.5 (1.5)	35.0 (29.1)
15	37	Treatment	Setup	Head not at correct treatment height for multiple fx (large change > 5 cm)	Incorrect dose delivered	1.4 (0.5)	7.0 (2.0)	3.0 (3.5)	29.4 (37.0)
16	63	Treatment	Delivery	Forget to flip to supine after prone field	Incorrect dose delivered	2.0 (1.1)	6.8 (1.7)	3.6 (3.4)	49.0 (54.7)
17	32	Treatment	Setup	Treatment head in wrong rotational position, not locked	Incorrect dose delivered	1.6 (0.8)	6.8 (1.6)	1.4 (0.5)	15.2 (10.0)
18	43	Treatment	Setup	Wrong flattening filter inserted	Incorrect dose delivered	1.0 (0.0)	6.3 (2.6)	4.3 (4.0)	27.4 (27.9)
19	73	QA	Machine QA	Incorrect calibration	Incorrect dose delivered	2.3 (1.2)	6.3 (2.6)	1.3 (0.5)	19.7 (15.0)
20	70	Treatment	Delivery	Treatment paused but not restarted	Incorrect dose delivered	1.8 (0.7)	6.2 (1.7)	3.6 (3.3)	40.2 (41.6)

Rank	FM#	Process description	Step Description	Failure mode	Effects	O	S	D	RPN
**C: D F B W—F M E A (6 scorers: 2 MPs, 2 ROs, 2 RTs) ranked wrt mean RPN scores**									
1	11	Planning	Preparation of initial plan	Corrupt spreadsheet	Incorrect dose calculation	1.8 (0.7)	7.0 (1.2)	5.8 (2.3)	74.9 (42.3)
2	51	Treatment	Delivery	Neglect to subtract in-vivo delivery time from remaining tx time	Incorrect dose delivered	2.5 (0.9)	6.5 (1.7)	4.0 (1.9)	65.0 (41.3)
3	16	Treatment	Setup	Setup error (significant wrt tx field)	Incorrect dose delivered	2.5 (1.6)	6.5 (2.1)	3.8 (2.6)	62.3 (61.6)
4	49	Treatment	Delivery	Program wrong tx time	Incorrect dose delivered	2.5 (0.8)	7.0 (1.8)	3.5 (1.7)	61.3 (38.7)
5	14	Planning	Ancillary	Incorrect compensator placement	Incorrect dose delivered	2.3 (0.4)	6.3 (1.3)	4.0 (1.9)	56.3 (30.8)
6	8	Planning	Preparation of initial plan	Calculation error	Incorrect dose calculation	2.0 (0.6)	7.2 (0.9)	3.8 (2)	54.9 (33.9)
7	9	Planning	Preparation of initial plan	Incorrect treatment date	Incorrect dose calculation	2.2 (0.7)	6.3 (1.4)	4.0 (2.3)	54.9 (38.1)
8	6	Planning	Preparation of initial plan	Miscommunication of Rx	Incorrect dose or fractionation	2.2 (0.4)	7.2 (1.1)	3.5 (1.9)	54.3 (31.9)
9	10	Planning	Preparation of initial plan	Incorrect documentation in EMR	Incorrect plan/dose delivered	2.2 (0.4)	6.2 (2.5)	4.0 (2.5)	53.4 (41.2)
10	52	Treatment	Delivery	Timer malfunction	Incorrect dose delivered	1.7 (0.5)	7.7 (1.6)	3.8 (2.0)	49.0 (30.3)
11	2	Logistics	Plan approval	Plan changed after checks but before approval on tx day	Incorrect plan delivered	2.5 (0.8)	5.3 (2.9)	3.7 (2.1)	48.9 (41.1)
12	40	Treatment	Setup	Flattening filter partially inserted	Incorrect dose delivered	1.8 (0.4)	6.7 (1.8)	3.8 (1.6)	46.9 (24.9)
13	41	Treatment	Setup	Flattening filter inserted but not latched	Incorrect dose delivered	1.8 (0.4)	5.7 (1.9)	4.5 (2.1)	46.8 (28.1)
14	13	Planning	Ancillary	Incorrect compensator	Incorrect dose delivered	2.0 (0.0)	6.5 (1.1)	3.5 (2.1)	45.5 (27.9)
15	7	Planning	Preparation of initial plan	Incorrect documentation	Incorrect dose calculation	2.0 (0.0)	7.2 (0.9)	3.2 (1.9)	45.4 (27.3)
16	1	Logistics	Treatment calendar	Miscommunication of dates	Plan not ready on tx date, plan calculated for wrong tx date	3.2 (1.0)	4.0 (2.4)	3.5 (1.4)	44.8 (35.4)
17	43	Treatment	Setup	Wrong flattening filter inserted	Incorrect dose delivered	1.5 (0.5)	6.8 (1.7)	4.2 (2.2)	42.7 (28.6)
18	22	Treatment	Setup	Wrong compensator	Incorrect dose delivered	2.0 (0.7)	6.0 (1.6)	3.5 (2.1)	42 (30.9)
19	53	Treatment	Delivery	Patient moves after setup (significant move wrt tx field) (no lung blocks)	Incorrect dose delivered	2.8 (0.9)	5.5 (2.0)	2.7 (0.9)	41.6 (24.8)
20	17	Treatment	Setup	Wrong lung blocks	Incorrect dose delivered	2.2 (0.4)	5.0 (2.0)	3.8 (2.3)	41.5 (30.5)

Rank	FM#	Process description	Step Description	Failure mode	Effects	O	S	D	RPN
**D: D F B W—F M E A (6 scorers: 2 MPs, 2 ROs, 2 RTs) ranked wrt mean ‘S’ scores**									
1	47	Treatment	Delivery	Wrong patient	Incorrect dose delivered	1.2 (0.4)	8.7 (0.9)	2.7 (1.4)	27.0 (16.6)
2	56	Treatment	Delivery	Source stuck	Incorrect dose delivered / staff exposure	1.2 (0.4)	8.7 (1.1)	1.3 (0.5)	13.5 (6.6)
3	79	QA	Machine QA	Source stuck and emergency shutter not operational	Patient overdose	1.2 (0.4)	8.3 (2.2)	2.2 (1.5)	21.1 (16.7)
4	42	Treatment	Setup	Flattening filter falls on patient	Patient injury	1.5 (0.5)	8.2 (0.9)	2.3 (2.1)	28.6 (28.0)
5	45	Treatment	Setup	Motor run away causing head to contact patient	Patient injury	1.2 (0.4)	8.0 (2.0)	1.5 (0.8)	14.0 (9.1)
6	52	Treatment	Delivery	Timer malfunction	Incorrect dose delivered	1.7 (0.5)	7.7 (1.6)	3.8 (2.0)	49.0 (30.3)
7	25	Treatment	Setup	Compensator falls on patient	Patient injury	1.5 (0.5)	7.5 (1.5)	2.8 (3.0)	30.9 (36.2)
8	8	Planning	Preparation of initial plan	Calculation error	Incorrect dose calculation	2.0 (0.6)	7.2 (0.9)	3.8 (2.0)	54.9 (33.9)
9	6	Planning	Preparation of initial plan	Miscommunication of Rx	Incorrect dose or fractionation	2.2 (0.4)	7.2 (1.1)	3.5 (1.9)	54.3 (31.9)
10	7	Planning	Preparation of initial plan	Incorrect documentation	Incorrect dose calculation	2.0 (0.0)	7.2 (0.9)	3.2 (1.9)	45.4 (27.3)
11	28	Treatment	Setup	Couch not at treatment height for multiple fx (large change > 5 cm)	Incorrect dose delivered	1.8 (1.1)	7.2 (2)	2.8 (2.1)	37.2 (36.8)
12	11	Planning	Preparation of initial plan	Corrupt spreadsheet	Incorrect dose calculation	1.8 (0.7)	7.0 (1.2)	5.8 (2.3)	74.9 (42.3)
13	49	Treatment	Delivery	Program wrong tx time	Incorrect dose delivered	2.5 (0.8)	7.0 (1.8)	3.5 (1.7)	61.3 (38.7)
14	21	Treatment	Setup	Lung blocks/tray fall on patient	Patient injury	1.2 (0.4)	7.0 (1.5)	2.0 (2.2)	16.3 (19.3)
15	19	Treatment	Setup	Missing lung blocks (all fractions)	Incorrect dose delivered	1.2 (0.4)	7.0 (1.8)	1.5 (0.8)	12.3 (8.0)
16	43	Treatment	Setup	Wrong flattening filter inserted	Incorrect dose delivered	1.5 (0.5)	6.8 (1.7)	4.2 (2.2)	42.7 (28.6)
17	39	Treatment	Setup	Flattening filter not inserted	Incorrect dose delivered	1.8 (0.4)	6.8 (2.0)	3.0 (1.4)	37.6 (22.3)
18	63	Treatment	Delivery	Forget to flip to supine after prone field	Incorrect dose delivered	1.7 (0.5)	6.8 (0.7)	2.2 (1.3)	24.7 (17.0)
19	40	Treatment	Setup	Flattening filter partially inserted	Incorrect dose delivered	1.8 (0.4)	6.7 (1.8)	3.8 (1.6)	46.9 (24.9)
20	74	QA	Machine QA	Incorrect beam data	Incorrect dose delivered	2.0 (0.6)	6.7 (1.8)	2.7 (1.1)	35.6 (20.4)

A number of preventative measures were implemented as a result of this FMEA. Table 2 lists the highest scoring FMs by RPN along with associated preventative measures. Of the 40 measures listed, 11 represent new interventions implemented as a result of this FMEA (5 at KCI and 6 at DFBW).

**Table 2:** Interventions resulting from the FMEA process

Rank	Failure mode	Preventative measure
A: K C I—F M E A		
1	Patient moves after setup (with lung blocks)	**Increased attentiveness to patient motion—one therapist assigned solely to monitoring patient motion**
2	Setup error (significant wrt tx field)	*Check of treatment field *via* light field visualization prior after setup and prior to treatment*
3	Calculation error	*Second check of calculation by physicist*
4	Couch is accidentally shifted after morning QA (shift insignificant wrt tx field)	**Therapists check couch location again after patient setup and prior to treatment**
5	Program wrong tx time	*Time out and double check by second therapist*
6	Patient moves after setup (significant move wrt tx field) (no lung blocks)	**Increased attentiveness to patient motion—one therapist assigned solely to monitoring patient motion**
7	Used prone time for supine position or vice versa	*Time out and double check by second therapist*
8	Timer malfunction	**Set stopwatch as secondary timer for all multi-fraction cases**
9	Missing lung blocks (one fraction)	*Time out and double check by second therapist*
10	Incorrect documentation (patient positioning)	*Measurements rechecked during setup on each treatment day*
11	Incorrect lung block design/size	*Evaluation of lung block size and shape prior to first treatment*
12	Forget to flip to supine after prone field	*Time out and double check by second therapist*
13	Incorrect mx	*Measurements rechecked during setup on each treatment day*
14	Corrupt spreadsheet	*Checked against manual calculation for all treatment fields*
15	Image from wrong date is used for alignment verification	*Lung blocking evaluated against anatomy on the current blocked image*
16	Patient has too many blankets covering them	*Checked prior to treatment*
17	Incorrect documentation (patient measurements)	*Measurements rechecked during setup on each treatment day*
18	Couch is accidentally shifted after morning QA (shift significant wrt tx field)	**Therapists check couch location again after patient setup and prior to treatment**
19	Images saved under wrong patient	*Lung blocking evaluated against anatomy on the current blocked image*
20	Miscommunication of Rx	*Physicist checks BMT physician’s treatment note in addition to RO physician’s treatment note*
B. D F B W – F M E A		
1	Corrupt spreadsheet	*Calculation performed by both a commissioned Java application and a spreadsheet*
2	Neglect to subtract in-vivo delivery time from remaining tx time	*Time out and double check by second therapist*
3	Setup error (significant wrt tx field)	*Check of treatment field *via* light field visualization after setup and prior to treatment*
4	Program wrong tx time	*Time out and double check by second therapist*
5	Incorrect compensator placement	*Compensator placement is checked before treatment with MV imaging*
6	Calculation error	*Two independent calculations are performed, one by dosimetry one by physics*
7	Incorrect treatment date	*Correct dates are second checked during physics check and therapist plan check*
8	Miscommunication of Rx	*Physics 2nd check compares planned dose to prescription* *Therapist plan check compares planned dose to prescription*
9	Incorrect documentation in EMR	*Fractionation checked against a protocol list*
10	Timer malfunction	*Secondary timer used during all treatments*
11	Plan changed after checks but before approval on tx day	*Timeout compares planned dose to prescription on tx day*
12	Flattening filter partially inserted	**Flattening filter position checked during morning QA**
13	Flattening filter inserted but not latched	**Flattening filter position checked during morning QA**
14	Incorrect compensator	**Compensators are checked against plan during therapist plan check**
15	Incorrect documentation	*Plan documentation checked during physics check and therapist plan check*
16	Miscommunication of dates	*Correct dates are second checked during physics check and therapist plan check*
17	Wrong flattening filter inserted	*The console will interlock if the wrong flattening filter is inserted and distance from the floor to the flattening filter would fail QA if wrong filter was in place*
18	Wrong compensator	**Compensators are checked against plan during therapist plan check**
19	Patient moves after setup (significant move wrt tx field) (no lung blocks)	**Increased attentiveness to patient motion—one therapist assigned solely to monitoring patient motion**
20	Wrong lung blocks	**Lung blocks are checked against plan during therapist plan check**

Bold—new intervention resulting from the FMEA; italics—existing QA/QC measure

Table 3 presents a variety of parameters that highlight the similarities and differences in various CPs. The number of common FMs (A∩B) in the top 20 ranked FMs ranged between 6 and 13, resulting in JI values of 18–48% respectively. AGD values as high as 407.3 were observed, whereas the mean and median AGD in any CP varied from 12.5 to 74.5, and 9.3 to 38.4 respectively.

**Table 3:** Similarities and differences for different comparison pairs (CPs)

CP	CP description	n (no. of FMs with full data)	For top 20 FMs (ranked w.r.t. RPN)		For ‘n’ number of FMs		
			A ∩ B	JI (%)	Range of AGDs	Mean AGD (SD in brackets)	Median AGD
1	KCI versus DFBW	87	6	18	0.1–69.6	14.6 (12.7)	11.9
2	MP versus RO	87	10	33	0.5–162.5	36.3 (34.1)	25.8
3	RO versus RT	87	10	33	0.2–48.1	12.5 (10.8)	9.8
4	RT versus MP	87	9	29	0.8–173.9	41.2 (37.9)	29.5
5	KCI- versus DFBW-MP	86	9	29	0.0–322.5	58.3 (68.8)	33.8
6	KCI- versus DFBW-RO	78	7	21	0.0–76.5	16.4 (17.3)	10.6
7	KCI- versus DFBW-RT	78	6	18	0.5–116.3	18.0 (18.7)	11.5
8	KCI-MP versus -RO	78	13	48	0.0–385.0	62.9 (75.2)	25.1
9	KCI-RO versus -RT	71	10	33	1.0–85.0	22.2 (18.7)	16.0
10	KCI-RT versus -MP	78	10	33	1.0–407.3	74.5 (85.7)	38.4
11	DFBW-MP versus -RO	86	7	21	0.4–120.0	26.1 (25.6)	16.8
12	DFBW-RO versus -RT	86	9	29	0.0–107.3	13.6 (14.9)	9.3
13	DFBW-RT versus -MP	86	6	18	0.0–132.0	26.1 (28.7)	15.4

As shown in Table 1, the two institutions have six common FMs in the top 20 ranked FMs (resulting in a JI of 18%) which is the lowest among all CPs. The inter-institutional intra-professional comparison shows that the number of common FMs in the top 20 list is the highest for MPs (9/20; JI = 29%), followed by ROs (7/20; JI = 21%) and RTs (6/20; JI = 18%). If we compare different specialties without being institution-specific, the number of common FMs in the top 20 is the same for MP vs RO and RO vs RT (10/20; JI = 33% each) and is slightly lower for RT vs MP (9/20; JI = 29%). Further, the JI values for inter-professional comparison differ from institution-to-institution as we evaluate KCI (CP 8, 9, 10 in Table 3) and DFBW (CP 11, 12, 13 in Table 3) data. For KCI, the number of common FMs is the highest for MPs vs ROs (13/20; JI = 48%) followed by ROs vs RTs and RTs vs MPs who both have 10/20 common FMs (JI = 33%). Whereas, for DFBW, the results show 9/20 common FMs for ROs vs RTs (JI = 29%) followed by MPs vs ROs with 7/20 (JI = 21%) and RTs vs MPs with 6/20 (JI = 18%).

The mean inter-institutional AGD value of 14.6 (SD:12.7) is smaller than that observed in either inter-professional comparisons or intra-professional comparisons across institutions. The overall inter-professional comparison (CP 2, 3, 4 in Table 3) suggests that there is a better agreement among ROs and RTs (mean AGD 12.5, SD: 10.8, range: 0.2–48.1) as compared to the other two CPs. This trend is the same even if the data from only one institution (whether KCI or DFBW) are observed.

Evaluation of the reproducibility of the FMEA results by the KCI physicists following a 2 year time interval revealed differences in RPN (new–old) ranging from + 55 to − 276 with a mean value of − 33. Despite this variability, 62 of the 87 FMs had a new RPN within ± 50 of the old RPN, and 16 of the top 20 FMs from the original FMEA were still in the top 20 in the new FMEA. These results demonstrate a small decrease in RPN over time but a relative consistency in the FMEA ranking as a function of time.

## Discussion

The results show that a multi-disciplinary, bi-institutional FMEA helps elucidate differences in processes between institutions as well as the prioritization of risks amongst different professional groups. Even when minor operational differences exist, institutions can benefit from mutual experiences, differing experiences, and resultant risk estimation. These operational differences represent the primary reason for the incomplete ratings for some FMs, although these incomplete ratings can still be used for analysis when at least one scorer’s rating is available. The dose calculation and in-vivo dosimetry processes represent an example of the influence of operational differences on FMEA ranking results. While both institutions make use of both a manual and a spreadsheet calculation for treatment time, KCI performs a second check of the manual calculation and performs in-vivo dosimetry for all patients. While corruption of the treatment time calculation spreadsheet is the highest ranking FM for DFBW, it is only the 15th highest for KCI, largely due to the fact that the detectability estimated by KCI is lower by almost a factor of three. While the rankings should not consider QA/QC measures in place, these existing measures may have unintentionally influenced the detectability ranking. Additionally, O and D scores are to be based on checks inherent in routine clinical processes downstream and some raters may have considered redundancies in treatment time calculation and/or in-vivo dosimetry to be routine clinical processes. This highlights one of the complexities involved in the implementation of an FMEA, particularly one which includes multiple institutions, and likely explains the large differences in estimated detectability for this FM.

The MPs tend to rate the greatest number of FMs even in the case of FMs related to bi-institutional operational differences. In contrast, ROs tend to omit rankings for FMs related to QA. Interestingly, some of the RTs also omitted rankings for some of the FMs related to QA as well as treatment setup. This indicates a potential lack of awareness of some safety and QA processes and/or risks by some groups or individuals involved in the treatment process. The fact that these individuals were included in the FMEA process is a valuable first step in recognizing these risks. In addition, the interventions developed to address the highest scoring FMs were shared among all groups participating in the treatment process, thus promoting further awareness of safety and QA processes and potential risks.

As shown for the individual indices, all scorers were inclined to rate ‘S’ more highly as compared to ‘O’ and ‘D’. This trend remains the same for both institutions and all specialties. This suggests either that participants consider the severity of the identified FMs as the most important factor or that the rating scale utilized from the AAPM TG-100 report favors higher ‘S’ scores, followed by difficulty in detectability, and occurrence of the events.

Furthermore, the results shown by Fig. 1 and Table 1 reveal high SD values compared to the mean values for individual indices (O, S, D). This highlights the fact that each FM can be rated quite differently by different scorers. This disparity is even higher for RPN values where SD is sometimes approximately double the mean value. This is because the SD in RPN is based on the propagation of the uncertainties in the factors used in calculating RPN (O, S, D).

It is observed that the highest RPN in the DFBW-FMEA is lower than that of the KCI-FMEA (104.9, SD:72.0 vs 74.9, SD:42.3), owing to inter-interinstitutional differences. The inter-professional comparison shows that MPs have the highest overall mean RPN value (192.5, SD:65.1) followed by ROs (70.1, SD:48.6) and RTs (63.4, SD:35.4). While O, S, and D mean scores were all higher for MPs, the largest differences between the MPs and other groups were in the scoring of occurrence and detectability. If the MP, as the group typically leading safety and QA, is the most knowledgeable about these risks, this result potentially indicates a greater sense of false security in the relative occurrence and detectability of FMs studied here. For the FMs with the most severe consequences, the different specialties tend to agree on ratings. ROs assigned the highest S-rating of 10 (SD:0.0), followed by MPs with 9.3 (SD:0.9) and RTs with 9.00 (SD:1.0). Likewise, for the frequency of occurrence of FMs, the highest mean O-rating was recorded by MPs (5.5, SD:1.5), followed by RTs (5.0, SD:2.0) and ROs (4.3, SD:1.8). Interestingly, participants with different specialties tend to disagree the most on the detectability (D-index). This is potentially due to a lack of familiarity with all mechanisms used to assure safety and quality and the participation of all groups in this FMEA is a positive step toward larger involvement of all groups in the safety process and increased vigilance for the identification of potential errors and unsafe practices. The overall mean value for MPs was 8.3 (SD:1.7) in comparison to 6.5 (SD:2.50) for RTs and 4.3 (SD:3.2) for ROs.

For the similarities in the top 20 FMs, the inter-professional similarity values (9 or 10 common FMs in top 20 i.e. 9–10/20; JI = 29–33%) are better than the inter-institutional similarity (6/20; JI = 18%). The inter-institutional intra-professional comparison shows that the MPs have the most intra-professional similarity (9/20; JI = 29%) compared to other specialties (ROs: 7/20 [JI = 21%], RTs: 6/20 [JI = 18%]). Intra-institutional inter-professional similarity shows that the trend in the similarity between specialties can vary from institution to institution. For KCI, JI_MP-RO_ = 48%, and JI_RO-RT_ = JI_RT-MP_ = 33% based on the number of common FMs in the top 20 i.e. 13/20, 10/20, and 10/20 respectively. Whereas, for DFBW, JI_MP-RO_ = 21%, JI_RO-RT_ = 29%, and JI_RT-MP_ = 18% for the number of common FMs as 7/20, 9/20, and 6/20 respectively. One might expect more commonality among the highest scoring FMs, however, this study serves to illustrate the potential differences, assumed primarily to be in the operational details, between institutions implementing the same procedures on the same equipment.

Evaluating AGD values from the FMEA, there is a good agreement in inter-institutional comparison (Mean AGD_KCI-DFBW_ = 14.6, SD:12.7) relative to the inter-professional comparisons (Mean AGD_MP-RO_ = 36.3, SD:34.1, AGD_RO-RT_ = 12.5, SD:10.8, AGD_RT-MP_ = 41.2, SD: 37.9). Interestingly, the AGD values in inter-institutional intra-profession comparison illustrate that the MPs have a greater difference (AGD = 58.3, SD = 68.8) as compared to ROs (AGD = 16.4, SD = 17.3) and RTs (AGD = 18.0, SD = 18.7). In addition, the inter-professional variability was found to be institution-specific.

A maximum AGD value of 407.3 was observed for FM#49 for KCI-RT ([O, S, D] = [1.5, 8.5, 1.0] and RPN = 12.5) vs KCI-MP ([O, S, D] =  [7, 10, 6] and RPN = 420). This FM was “Programming the incorrect treatment time.” This very large discrepancy highlights the differences in estimation of O, S, and D values across professions. Both RTs and MPs evaluated this FM as relatively severe (S = 8.5 and 10 for RTs and MPs, respectively). However, RTs estimated an occurrence frequency between 0.01 and 0.02%, compared to 0.5–1% for MPs, and RTs estimated a detectability of 99.99%, compared to 95% for MPs. So one may question whose risk estimations are more accurate, the group who oversees departmental quality, safety, and risk assessment, or the group who is actually performing this function. If we had adequate data available to answer this question, we wouldn’t necessarily need to apply prospective risk assessment. Instead, these differences help us understand the relative uncertainties involved in the prospective risk assessment process. Differences between groups in the estimation of occurrence and detectability for this FM result in a relative increase in the overall RPN score by a factor of 4.7 and 6, respectively. Such large changes would substantially change the ranking of a FM. For example, dividing the highest aggregate RPN in our FMEA by a factor of 5 would demote it the 73rd highest ranking FM, thus dramatically reducing the attention it would receive as a potential risk factor. This highlights the importance of participation of as many individuals as possible in performing the prospective risk analysis and that it should not be restricted to individuals within a particular discipline with particular perspectives on the associated processes. This reinforces the TG-100 report recommendation to use “a team-based approach that requires active participation of representatives from all treatment team member categories” [3].

The differences observed between the original and new FMEAs performed by the KCI MP participants highlights the potential variability in the results of an FMEA, as the 2-year time difference was more than enough to assure that there was no recollection of the original FMEA scoring. However, it also likely represents changes in perceived occurrence, severity, and detectability over time. The average overall decrease in RPN over this 2-year period likely represents an increased comfort level that FMs are either less likely to occur (for those that have not yet occurred) or more likely to be detected (due to greater experience with the treatment unit), or both. A similar result was obtained by Mancosu et al. (2021) for repetition of an FMEA after 10 years (25). In those 10 years, there has been a great deal of evolution in the performance of the FMEA in radiation oncology.

One should be very careful in applying AGDs for comparison, whether between institutions or among different specialties. Two FMs with different O, S, and D index values may end up with comparable RPN values thus showing lower AGD values [25]. Similarly, the data on JI is confined to 20 FMs in our case (for the first stage/high priority). Therefore, we recommend that the results on AGD should be used in conjunction with JI for the top 20 FMs to draw any conclusions.

Also, the intra- or inter-institutional and -professional comparisons of the FMs ranked with respect to the individual index values may not be a good approach as such a ranking is highly subjective. As an example, the DFBW-FMEA suggests that the most frequent FM is the therapist hurting themselves while trying to move the patient. In contrast, this FM is ranked 66th most frequent in KCI-FMEA. Such differences could arise purely from the past experiences of one institution or profession.

There is often a discordance between a clinician’s “gut feeling” about the relative risk associated with an event, and a formal FMEA evaluation of that event. As an example, a source stuck in the exposed position accompanied by failure of the emergency shutter to deploy sounds like a catastrophic event. However, there are abundant detection mechanisms for this event inherent in the downstream clinical processes. As such, this failure would be detected relatively quickly even without the consideration of QA/QC measures (as required in the TG-100 FMEA). Estimating the time associated with routine detection and subsequent time for remediation based on our emergency procedures and mock drills, we anticipate that such an event will most likely result only in a minor dosimetric error to the patient. Even assuming a worst case-scenario for severity, the very low predicted occurrence of both the source getting stuck and the emergency shutter failing simultaneously combined with the very high detectability of this event result in an RPN score that is relatively modest.

In other industries, a cut-off RPN value (or of individual indices) is often determined (instead of a prioritization value) [26] to select a handful of FMs for consideration. In those industries, the preventive measures are suggested for that set of FMs and the rest may be discarded. We do not consider any FMs as irrelevant in radiation oncology. On the other hand, prioritizing only a small set of FMs for intervention helps these changes to be translated into the department relatively simply and without major changes [3]. We chose a small representative set for the initial stage of the top 20 ranked by RPN, or 1/4 of the total number of FMs. Of course, a different number can be chosen depending on the human resource availability and readiness of the staff for a change in QM.

## Conclusion

We present the first bi-institutional multi-disciplinary FMEA analysis of a Co-60 based dedicated TBI technique. The results highlight the relative variability in ranking FMs when sampled across multiple professions and institutions. Differences in ratings at the two institutions were often linked with operational differences. The inter-professional variability appears to be institution-specific. We find that a bi-institutional and/or multi-disciplinary FMEA is not only feasible but is also helpful in many ways. Such a comparison allows the institutions to learn from one another’s processes, experiences, and risk estimates in seeking areas for improvement in their QM program. Similarly, the inter-professional comparison helps evaluate differences in risk estimation among different specialties. Such variability may be due to either difference in processes or differences in perceptions of risk between institutions and professions. It ultimately helps both in the identification of the most important FMs and in identifying the best practices to address those FMs. The involvement of all groups participating in the treatment process within an FMEA not only improves the quality of data obtainable from the FMEA, but also should have a tangible benefit on departmental QA and safety. Providing all stakeholders with a better understanding of potential risks and their relative occurrence, severity, and detectability, promises to help maximize quality assurance, minimize risks, and improve the overall safety culture of the department.

## Supplementary Information

**Additional file 1.** Aggregate FMEA (the mean FMEA ranked by all participants from both institutions).

## Data Availability

All data generated or analyzed during this study are included in this published article (and its Additional file 1).

## Abbreviations

TBI: Total body irradiation
FMEA: Failure mode and effects analysis
FM(s): Failure mode(s)
O: Occurrence
S: Severity
D: Detectability
RPN: Risk priority number
SD: Standard deviation
AGD: Absolute gross differences
JI: Jaccard Index
MP(s): Medical physicist(s)
RO(s): Radiation oncologist(s)
RT(s): Radiation therapist(s)
QM: Quality management
IMRT: Intensity-modulated radiotherapy
TMI: Total marrow irradiation
QA: Quality assurance
QC: Quality control
Aggregate-FMEA: An FMEA with ratings by all participants from both institutions
KCI: Karmanos Cancer Institute
DFBW: Dana-Farber/Brigham and Women’s Cancer Center
KCI-FMEA: An FMEA with ratings by all participants from KCI
DFBW-FMEA: An FMEA with ratings by all participants from DFBW
X-FMEA: An FMEA with ratings by participants with the same specialty (X, which can either be MP, or RO, or RT) from both institutions.
A-X-FMEA: The FMEA ratings from participants with the same specialty (X) and belong to the same institution (A, which can either be KCI or DFBW)
MoM: Mean of means
CP(s): Comparison pair(s)
